# Trauma Severity and Its Impact on Local Inflammation in Extremity Injury—Insights From a Combined Trauma Model in Pigs

**DOI:** 10.3389/fimmu.2019.03028

**Published:** 2020-01-09

**Authors:** Klemens Horst, Johannes Greven, Hannah Lüken, Qiao Zhi, Roman Pfeifer, Tim P. Simon, Borna Relja, Ingo Marzi, Hans-Christoph Pape, Frank Hildebrand

**Affiliations:** ^1^Department of Orthopedic Trauma, University Hospital Aachen, Aachen, Germany; ^2^Orthopedic Trauma Research Laboratory, University Hospital Aachen, Aachen, Germany; ^3^Department of Orthopaedic Trauma Surgery, University Hospital Zurich, Zurich, Switzerland; ^4^Department of Intensive Care and Intermediate Care, RWTH Aachen University, Aachen, Germany; ^5^Department of Trauma-, Hand- and Reconstructive Surgery, University Hospital Frankfurt, Frankfurt, Germany; ^6^Experimental Radiology, Department of Radiology and Nuclear Medicine, Otto von Guericke University Magdeburg, Magdeburg, Germany

**Keywords:** polytrauma, pigs, local inflammation, extremity, hematoma, muscle, fixation

## Abstract

**Background:** Extremity fracture is frequently seen in multiple traumatized patients. Local post-traumatic inflammatory reactions as well as local and systemic interactions have been described in previous studies. However, trauma severity and its impact on the local immunologic reaction remains unclear. Therefore, fracture-associated local inflammation was investigated in a porcine model of isolated and combined trauma to gain information about the early inflammatory stages.

**Material and Methods:** Polytrauma (PT) consisted of lung contusion, liver laceration, femur fracture, and controlled hemorrhage. Monotrauma (MT) consisted of femur fracture only. The fracture was operatively stabilized and animals were monitored under ICU-standard for 72 h. Blood, fracture hematoma (FH) as well as muscle samples were collected throughout the experimental period. Levels of local and systemic pro- and anti-inflammatory as well as angiogenetic cytokines were measured by ELISA.

**Results:** Both groups showed a significant decrease in pro-inflammatory IL-6 in FH over time. However, concentrations in MT were significantly higher than in PT. The IL-8 concentrations initially decreased in FH, but recovered by the end of the observation period. These dynamics were only statistically significant in MT. Furthermore, concentrations measured in muscle tissue showed inverse kinetics compared to those in FH. The IL-10 did not present statistical resilient dynamics over time, although a slight increase in FH was seen by the end of the observation time in the MT group.

**Conclusions:** Time-dependent dynamics of the local inflammatory response were observed. Trauma severity showed a significant impact, with lower values in pro- as well as angiogenetic mediators. Fracture repair could be altered by these trauma-related changes of the local immunologic milieu, which might serve as a possible explanation for the higher rates of delayed or non-union bone repair in polytraumatised patients.

## Introduction

Trauma severity directly affects the pattern of injuries. Beside injuries to the head, thorax and abdomen, extremity injuries are common, and present in the majority of multiple traumatized patients (1, 2). However, fracture incidence also increased in the non-polytraumatised patient population during the past decade (3). The severity of extremity injury and its negative impacts on long-term outcome are well-documented (4–6). While pain and limited range of motion are frequently seen in isolated trauma (4, 5), previous clinical and experimental studies linked multiple trauma to significantly longer fracture healing times and higher incidences of non-unions in comparison to isolated fractures (7–9). Overwhelming local and systemic inflammatory responses with an associated negative influence on downstream processes of bone repair are a potential pathomechanism for this impaired fracture healing (10–13). Despite knowledge about the connectivity between the systemic and local inflammatory responses, information on the impact of trauma severity on systemic and local immunologic interactions and responses is scarce (14). Against this background, the purpose of this study was to investigate and compare systemic and local inflammatory responses in isolated and combined trauma. Within an established long-term porcine model of combined trauma (femur fracture, chest-, and abdominal injury, and hemorrhagic shock) (15), post-traumatic immunologic responses were analyzed and compared to those gained from a group with isolated femur fracture. Early kinetics of systemic and local (fracture hematoma and surrounding muscle tissue) immunologic response around the fracture zone were investigated during a 72 h clinically realistic study period.

## Materials and Methods

### Animal Care

Official permission to perform the study was granted from the governmental animal care and use office (Landesamt für Natur, Umwelt und Verbraucherschutz Nordrhein-Westfalen, Recklinghausen, Germany, AZ: 84.02.04.2014A265). All experimental protocols were approved by the governmental animal care and use office and performed in accordance with the German legislation governing animal studies, following The *Principles of Laboratory Animal Care* (16). The data presented in this paper were collected in the context of a larger study (15) for the benefit of the principles of the 3Rs (Replacement, Refinement, and Reduction) (17).

In total 24 male pigs (German Landrace, *Sus scrofa*) weighing 30 ± 5 kg, aged 3 months were used. After arrival from a disease-free barrier breeding facility all animals underwent clinical examination by a veterinarian. Thereafter all animals were housed for 7 days before experiments started. Polytrauma (PT) was induced to 12 animals while 12 animals received isolated femur fracture and were defined as monotrauma (MT). Animals were housed in ventilated rooms and allowed to acclimatize to their surroundings for a minimum of 7 days before start of the experiment. All sections of this report adhere to the ARRIVE Guidelines for reporting animal research (18).

### Sample Size and Power Calculation

A sample size calculation was performed for the primary study (15). The chosen sample sizes of 12 in the two groups (MT, PT) show comparable effect sizes as observed in a previous published study on hypothermia in a porcine trauma model (19) and will provide at least 80% power at a significance level of 5%. As all physiological, morphological and inflammatory outcomes characterizing the long-term evolution of severe multiple trauma are equally important to describe the intermodal animal model, no distinction between primary, and secondary outcome was made.

### General Instrumentation and Anesthesia

The model was previously described in detail elsewhere (15). In brief: premedication was induced by an intramuscular injection of azaperone (4 mg kg^−1^). During the 12-h fasting period animals had free access to water. Anesthesia was induced by propofol (3 mg kg^−1^), followed by orotracheal intubation. Volume-controlled, lung protective mechanical ventilation was applied, and vital parameters were continuously monitored and documented as previously described (20).

During the entire study period, general anesthesia was maintained with propofol and sufentanil (40–90 μg Kg^−1^/h). Continuous crystalloid infusion (Sterofundin ISO® 2 ml/kg BW/h) preserved animals from dehydration (15).

Administration of fluids and anesthesia was done by a central venous catheter which was placed in the external jugular vein. Furthermore, this was used to monitor the central venous pressure. The right femoral vein was instrumented via a three-lumen haemodialysis catheter to perform hemorrhage. Continuous monitoring of blood pressure, e.g., mean arterial pressure (MAP) was performed via an arterial line, that was placed in the femoral artery. Reference for intravascular pressure measurements was the mid-chest level and at end of expiration. Suprapubic catheter was applied. Finally, random allocation to either the PT group or the MT group was performed (15).

### Induction of Multiple Trauma and Hemorrhage

Trauma was induced as previously described (15) and after achieving stable baseline conditions (at least 120 min after instrumentation). During the 90-min period of shock animals were not prevented from hypothermia to simulate the clinical situation (in humans) after trauma and transport to the hospital.

A bolt gun machine (Blitz-Kerner, turbocut JOBB GmbH, Germany) was used to induce femur fracture in mono- as well as multiple trauma. Therefore the bolt hit a custom-made punch positioned on the mid third of the femur. Cattle-killing cartridges (9 × 17; DynamitNobel AG, Troisdorf, Germany) were used. The PT group received blunt chest trauma, induced by a bolt shot fired on a pair of panels that was placed on the right dorsal, lower chest (20, 21). Lungs were inflated when the bolt shot was applied. Moreover, a midline-laparotomy was performed and the right upper liver lobe was explored in PT. A penetrating hepatic injury was induced by a crosswise incision (4.5 x 4.5 cm) halfway through the liver tissue (22, 23). Liver packing was carried out with five sterile packs of 10 × 10-cm gauze after a short period of uncontrolled bleeding (30 s). Thereafter, hemorrhagic shock was induced by withdrawal of blood until a MAP of 40 ± 5 mm Hg was reached, with a maximum withdrawal of 45% of the total blood volume. The MAP was maintained for 90 min. The ISS (Injury Severity Score) was calculated as 27 points in PT. One investigator (KH) induced trauma, and the period of shock was monitored by two experienced clinicians (KH, TPS) (15).

Animals were resuscitated at the end of the shock period in accordance with established trauma guidelines (ATLS®, AWMF-S3 guideline on Treatment of Patients with Severe and Multiple Injuries®) by adjusting FiO_2_ to baseline values and re-infusing the withdrawn blood and additional fluids (Sterofundin ISO® 2 ml kg/BW/h) in PT (24). Furthermore, animals were rewarmed until normothermia (38.7–39.8°C) was reached using a forced-air warming system (24).

According to established trauma guidelines, operative stabilization of the femur fracture was performed after surgical disinfection and sterile draping at the end of the resuscitation (25). Fluoroscopy (Ziehm Vision, ZiehmImaging, Germany) was used to guide reduction and operation of the femur fracture. According to the clinical situation were internal as well as external stabilization is used for fracture treatment, an intramedullary nail (T2 System, Stryker) was applied to six animals in each group while the remaining six animals received external fixation (Radiolucent Fixator, Orthofix). Surgery was performed by one experienced trauma surgeon. Before surgery and then every 24 h until the end of the experiment antibiotics (Ceftriaxon® 2 g, i.v.) were administered (15).

### Data Collection

Following parameters were measured every 30 min by blood gas analysis (BGA) for a period of 5.5 h after trauma: pH, lactate (LAC), pCO_2_, pO_2_, hemoglobin (Hb), and base excess (BE). From then on, BGA was performed every 6 h until observation period came to an end. Time points of whole blood sampling are paralleled by data on physiologic responses (MAP and heart rate; HR) as well as BGA results. Results demonstrating severe signs of shock were published earlier (15).

Blood samples used in this study were obtained after resuscitation and operative treatment (3.5 h) and after 24, 48, and 72 h (15). Samples were kept on ice. Subsequently, after centrifugation at 2,000 × g for 15 min at 4°C, serum samples were stored at −80°C until analysis of IL-6, IL-8, and IL-10 concentrations (Quantikine® porcine ELISA kit; R&D systems, USA), according to the manufacturer‘s instructions. Muscle tissue was obtained by biopsy after resuscitation and operative treatment (3.5 h) and after 24, 48, and 72 h; samples were frozen in liquid nitrogen. For protein analysis, 100 mg of frozen muscle tissue were thawed in 300 μL of lysis and extraction buffer and immediately homogenized in an Eppendorf tube on ice with a T10 basic ULTRA-TURRAX® (IKA, Germany). Protein concentrations were measured with commercially available ELISA kits. Fracture hematoma was extracted under sterile conditions by puncturing the fracture zone at 3,5, 24, 48, and 72 h. Hematoma was collected in an EDTA monovette® (SARSTEDT AG & Co, Germany) and diluted with Sterofundin® 1:1. After centrifugation, serum was removed and stored at −80°C for further analysis. Referring to higher concentrations, all fracture hematoma samples were diluted once more (IL-6 1:10, IL-8: 1:4, IL-10: 1:4).

### Statistical Analysis

Statistics were performed with SPSS (Version 21.0.0.0) using Mann-Whitney-U, Wilcoxon rank sum and Friedman tests (including Chi^2^-Test) [illustrated as mean (SEM)]. For all comparisons, the significance level was set at 5%. Graphics were created using SPSS.

## Results

### Physiological Response

In contrast to previously reported data from the PT group (15), MT did not present with comparable shock parameters. The mean arterial pressure (MAP) was significantly higher (*p* < 0.001) in MT (69 ± 2.3 mmHg) than in PT (43 ± 1.9 mmHg) 90 min after trauma induction. Additionally, heart rate was significantly lower (*p* < 0.001) in MT (85 ± 7b/min) compared to PT (170 ± 11b/min) at this time. Furthermore pH (MT 7.51 ± 0.01 vs. PT 7.43 ± 0.01, *p* < 0.001), Lactate (MT 1.2 ± 0.2 mmol vs. PT 4.4 ± 0.4 mmol, *p* < 0.001), Base Excess (MT 4.8 ± 0.5 mmol vs. PT 0.4 ± 0.6 mmol, *p* < 0.001) did prove severe haemorrhagic shock only in the PT group. In regard to the reported time points during further clinical course only Lactate was slightly increased after 3.5 h (MT 1.01 ± 0.12 mmol vs. PT 1.38 ± 0.11 mmol). Otherwise there were no statistically significant differences found between the groups. Due to interrupted warming during the trauma phase body temperature was 36.7 ± 0.3°C in MT and 36.7 ± 0.2°C in PT after 90 min. These values were not statistically significant (*p* = 0.887). After rewarming, animals presented with physiological body temperature (MT 3.5 h: 38.2 ± 0.2°C; D1: 38.7 ± 0.1°C; D2: 38.7 ± 0.3°C and D3: 38.8 ± 0.1°C, *p* < 0.001 resp. PT 3.5 h: 37.9 ± 0.1°C; D1: 38.7 ± 0.1°C; D2: 38.8 ± 0.1°C and D3: 38.9 ± 0.1°C, *p* < 0.001). Although temperature changed statistically significant during the clinical course in both groups, there were no differences between the groups.

### Interleukin-6

**Figure 1:**
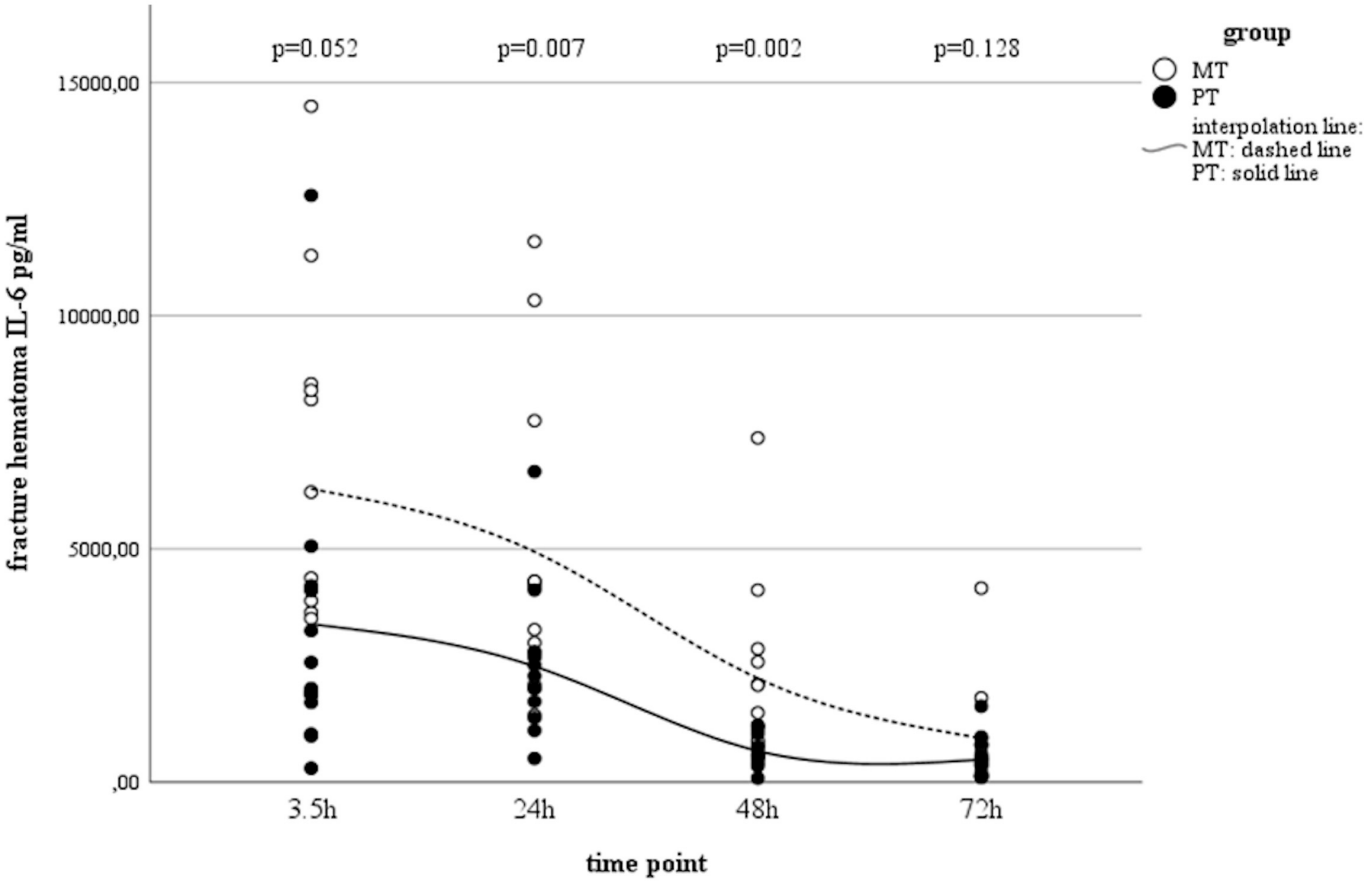
IL-6 concentrations in fracture hematoma between groups and different time points (PT, Polytrauma; MT, Monotrauma; pg/ml, picogram per milliliter; h, hours).

According to the post-traumatic phase, a decrease in serum concentrations was observed in PT, while concentrations in MT remained stable on a low level (Table 1). In both groups, a statistically significant decrease in local IL-6 concentrations in muscle tissue as well as fracture haematoma were observed over time (Table 1 and Figure 1). Although fracture haematoma concentrations were higher compared to serum concentrations in both groups, local concentrations of IL-6 in fracture haematoma were significantly lower in PT than in MT (Table 1). In contrast to PT, haematoma concentrations in MT showed statistically significantly higher levels compared to muscle tissue concentrations (Table 1).

**Table 1:** Systemic and local concentrations of IL-6 pg/ml; ^a^*p* < 0.05 compared to serum concentrations, ^b^*p* < 0.05 compared to muscle concentrations, ^c^*p* < 0.05 compared to PT.

**Time (h)**	**Polytrauma (PT)**			**Monotrauma (MT)**		
	**Serum**	**Muscle**	**Haematoma**	**Serum**	**Muscle**	**Haematoma**
3.5^W^	154 (24)	1,803 (535)	3,387 (927)^a^	65 (16)^c^	2,665 (377)^a^	6,286 (1,158)^a,b^
24^W^	81 (11)	1,244 (222)^a^	2,483 (463)^a^	56 (18)	1,347 (230)^a^	4,940 (932)^a,b,c^
48^W^	77 (19)	348 (42)^a^	671 (95)^a^	49 (24)	492 (120)^a^	2,228 (560)^a,b,c^
72^W^	63 (16)	170 (25)	485 (133)^a^	58 (20)	160 (54)	945 (318)^a,b^
*p*-value^F^	0.002	0.011	<0.001	0.858	0.001	<0.001

*Values are given in mean (SEM)*,

F *Friedman-Test and Chi^2^-Test*,

W *Wilcoxon-Test*.

### Interleukin-8

**Figure 2:**
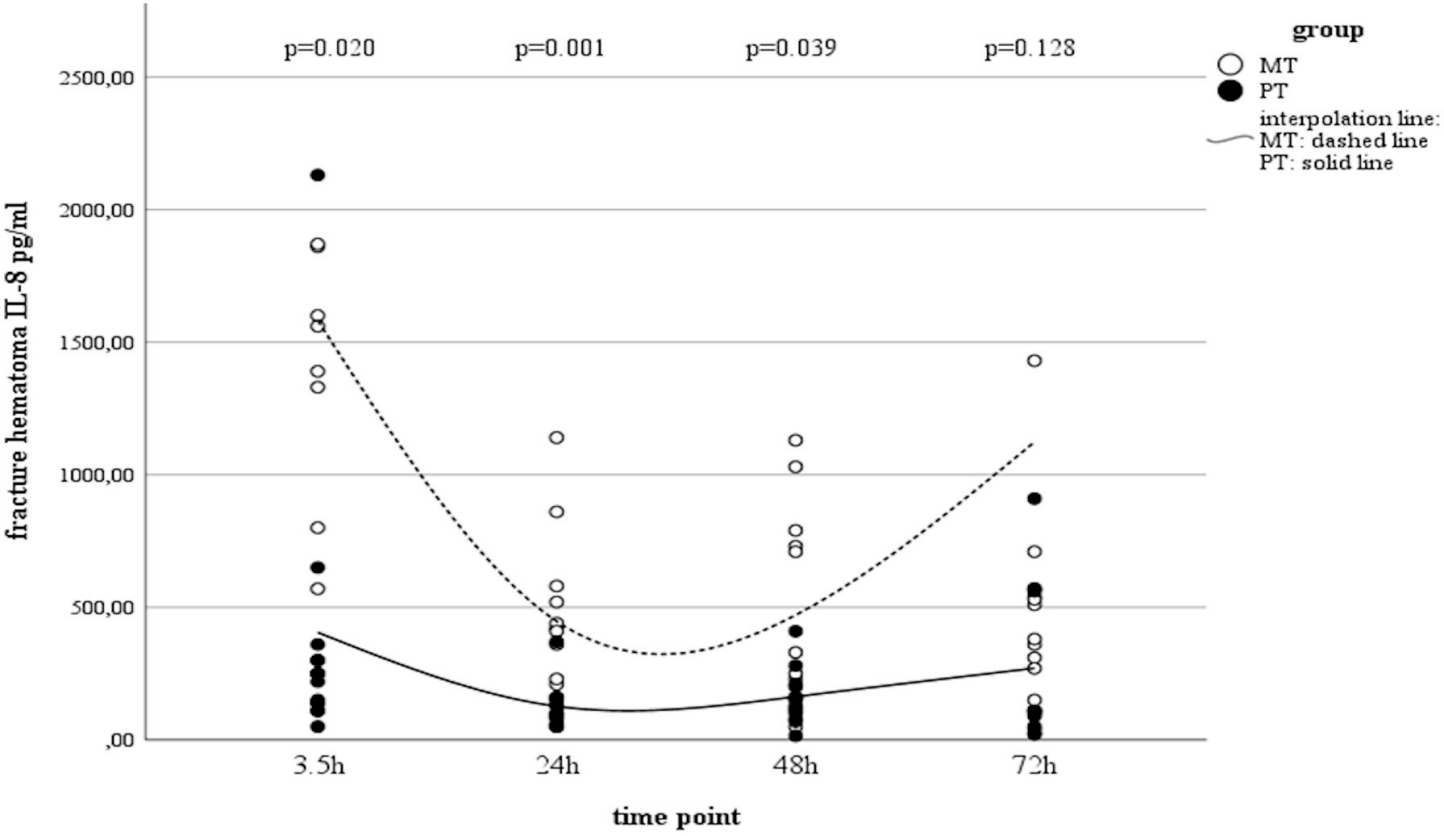
IL-8 concentrations in fracture haematoma between groups and different time points (PT, Polytrauma; MT, Monotrauma; pg/ml, picogram per milliliter; h, hours).

Serum IL-8 showed a slight increase over time in PT, while in MT, there was a decrease in systemic concentrations. However, this finding in MT was statistically not significant (Table 2). Interestingly, concentrations in muscle tissue showed opposite trends compared to the systemic ones. Initially increasing concentrations decreased by the end of the observation time (Table 2). In contrast, concentrations measured in fracture haematoma presented with inverse dynamics compared to those seen in muscle tissue. IL-8 dynamics in haematoma described a v-shaped curve, which was statistically significant in MT (Table 2 and Figure 2). At all measured time points, IL-8 concentrations in fracture haematoma of MT were higher compared to those in PT (Table 2).

**Table 2:** Systemic and local concentrations of IL-8 pg/ml; ^a^*p* < 0.05 compared to serum concentrations, ^b^*p* < 0.05 compared to muscle concentrations, ^c^*p* < 0.05 compared to PT.

**Time (h)**	**Polytrauma (PT)**			**Monotrauma (MT)**		
	**Serum**	**Muscle**	**Haematoma**	**Serum**	**Muscle**	**Haematoma**
3.5^W^	4 (1)	2,172 (400)^a^	405 (163)^a^	25 (11)	5,947 (2,116)^a^	1,582 (566)^a,b,c^
24^W^	11 (6)	8,115 (1,517)	127 (24)^a^	13 (5)	10,656 (2,116)^a^	448 (88)^a,b,c^
48^W^	12 (4)	6,704 (2,895)^a^	163 (30)^a,b^	12 (7)	9183 (2,711)^a^	471 (111)^a,b,c^
72^W^	13 (5)	1,782 (1,386)^a^	270 (86)^a^	15 (7)	555 (174)^a^	1,123 (658)^a^
*p*-value^F^	0.01	0.026	0.05	0.514	0.011	0.022

Values are given in mean (SEM),

F *Friedman-Test and Chi^2^-Test*,

W *Wilcoxon-Test*.

### Interleukin-10

**Figure 3:**
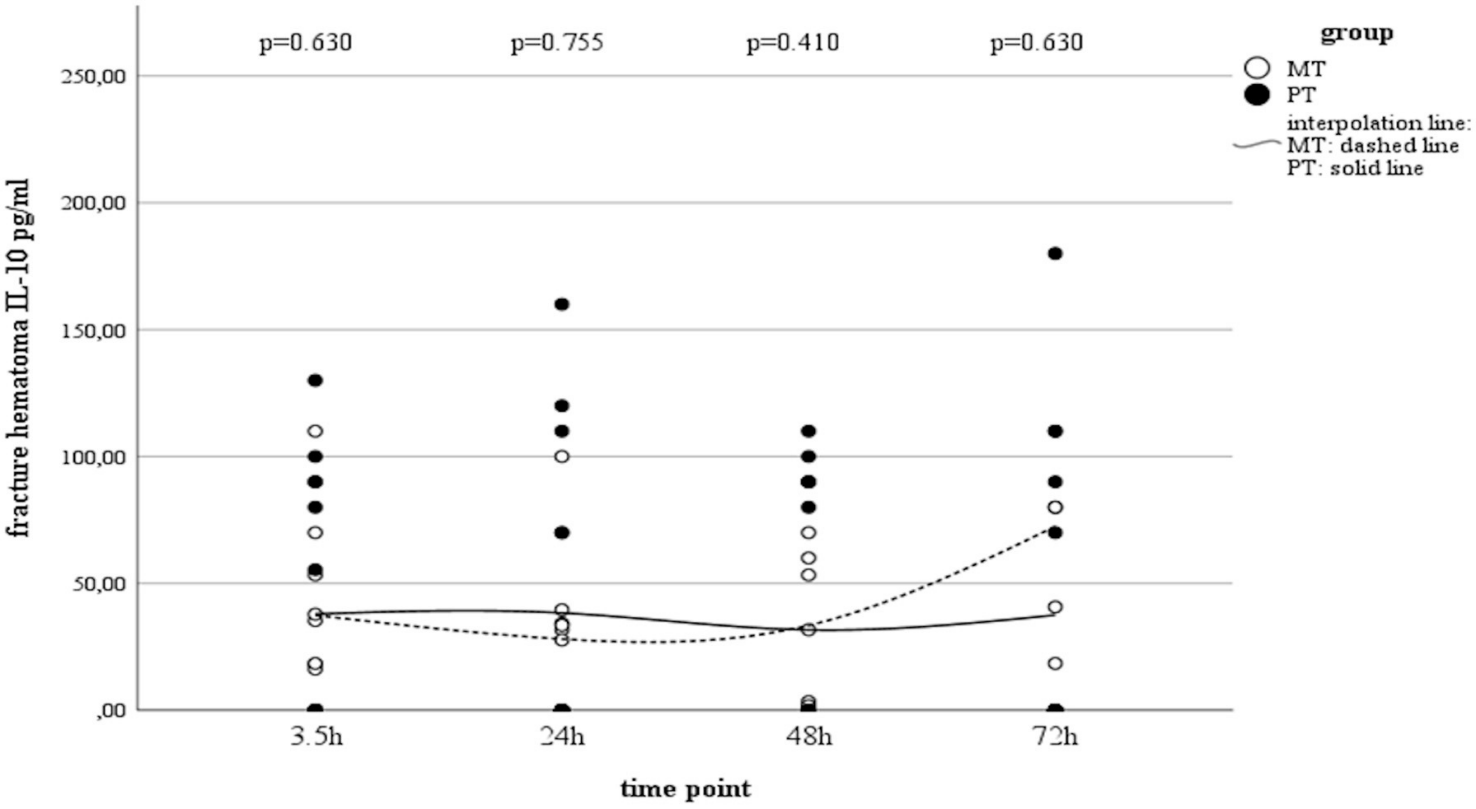
IL-10 concentrations in fracture haematoma between groups and different time points (PT, Polytrauma; MT, Monotrauma; pg/ml, picogram per milliliter; h, hours).

While serum concentrations of IL-10 in PT slightly decreased over time (Table 3), values in MT did not present statistically significant changes over time. Although local concentrations measured in muscle tissue and fracture hematoma remained uneventful and were detectable only on a very low level, haematoma concentrations in MT showed an increase by the end of the observation period (Table 3 and Figure 3). However, this finding was not statistically significant.

**Table 3:** Systemic and local concentrations of IL-10 pg/ml; ^a^*p* < 0.05 compared to serum concentrations, ^b^*p* < 0.05 compared to muscle concentrations, ^c^*p* < 0.05 compared to PT.

**Time (h)**	**Polytrauma (PT)**			**Monotrauma (MT)**		
	**Serum**	**Muscle**	**Haematoma**	**Serum**	**Muscle**	**Haematoma**
3.5^W^	48 (25)	0 (0)^a^	38 (14)	85 (47)	5 (5)	37 (11)^b^
24^W^	39 (26)	18 (7)	38 (17)	92 (50)	15 (10)	28 (9)
48^W^	33 (30)	12 (7)	32 (14)	145 (86)	25 (20)	33 (11)
72^W^	25 (21)	0 (0)	38 (18)	86 (45)	0 (0)	72 (44)^b^
*p*-value^F^	0.027	0.101	0.972	0.260	0.392	0.779

*Values are given in mean (SEM)*,

F *Friedman-Test and Chi^2^-Test*,

W *Wilcoxon-Test*.

## Discussion

Fracture healing is significantly influenced by the local inflammatory response after trauma (26–29). The impact of trauma severity may lead to a different post-traumatic response, which potentially influences the onset of fracture healing (30, 31). However, information about local inflammatory reactions regarding fracture repair are mostly gained from small animal models with either limited observation time or conditions that do not closely mimic a clinically realistic situation (32–36). As pigs respond to trauma similar to humans, we used an established long-term porcine model of isolated and multiple trauma to investigate the local and systemic inflammatory responses in regard to extremity injury and trauma impact (37, 38).

The main results might be summarized as follows:

- Local fracture haematoma concentrations of pro-inflammatory IL-6 and angiogenetic IL-8, but not of anti-inflammatory IL-10, exceeded the systemic values. Fracture haematoma concentrations of IL-6 and IL-8 were higher in MT compared to those in PT.
- In both groups, IL-8 concentrations in muscle tissue showed contrary dynamics compared to those seen in fracture haematoma. Concentrations in muscle tissue exceeded haematoma concentrations. Dynamics of haematoma concentrations described a v-shaped curve, implying a temporary decrease before recovery. This trend was statistically significant only in MT.
- Anti-inflammatory IL-10 presented increasing concentrations in fracture haematoma of MT, but not in PT by the end of the observation period, demonstrating a shift toward an inflammatory milieu. However, this finding was not statistically significant.

### The Pro-inflammatory Phase

The early post-traumatic immunologic milieu of fracture hematoma is characterized by inflammation and hypoxia (28). During this early period of acute inflammation, pro-inflammatory mediators such as IL-6 recruit cells needed for tissue regeneration (39). As previously reported and confirmed by others, IL-6 in fracture haematoma increases during the initial post-traumatic phase, followed by a continuous decrease during the further clinical course (20, 40, 41). While its early peak is discussed to maintain the onset of bone healing, persistent high values negatively influence osteogenic differentiation from stem cells (42–44). In regard to multiple trauma commonly associated with an advanced post-traumatic inflammatory response (45), Recknagel et al. revealed that concomitant thoracic trauma considerably enhanced the number of PMN, decreased the number of macrophages and slightly increased IL-6 expression locally at the fracture site, suggesting that post-traumatic systemic inflammation altered the finely tuned inflammatory balance during the early healing phase, leading to impaired bone healing (31, 46). Accordingly, De Benedetti et al. showed that overexpression of IL-6 resulted in severe osteopenia with reduced osteoblast and increased osteoclast numbers and activity (47). Thus, the observed time-dependent decrease in IL-6 concentrations seems to be a consistent step in the sequence of fracture repair. Heiner et al. suggested IL-6-induced up-regulation of the suppressor of cytokine signaling-3 (SOCS-3) as a possible mechanism for the reduction of local IL-6 concentrations (40).

Although we found this decrease in both groups, significant differences between concentrations of IL-6 in MT and PT were observed, with higher values in MT. This dichotomy is interesting as excessive trauma is known to increase systemic cytokine concentrations (14). However, in contrast to lung contusion or haemorrhagic shock, fracture associated soft tissue trauma was found not to be the driving force leading to significant increase of cytokine concentrations (36, 48, 49). Moreover, haemorrhagic shock was discussed to reduce supply in the fracture zone (50), which might explain the observed lower cytokine concentrations in PT fracture hematoma compared to MT ones. Altered immunologic reactions after bone injury with hemorrhage compared to isolated bone injury were previously described and support our findings (51–53). An altered immunologic milieu in the early fracture hematoma also support the findings reported by Lichte et al. who demonstrated impaired bone healing and a significantly decreased number of osteoclasts, a decrease in bone quality and more cartilage islands after hemorrhagic shock in mice (53). Additionally, Wichmann et al. reported on a murine model comparing isolated tibia fracture with tibia fracture and combined hemorrhagic shock (51). The authors concluded that severe hemorrhage after closed bone fracture depresses osteoblast activity and increases osteocyte necrosis, which should compromise fracture healing under those conditions (51). In line with others, the authors discussed decreased blood supply to the fracture zone to negatively influence fracture repair (51–53). Thus, our observation of lower pro-inflammatory concentrations in the PT group suggests the absence of important pro-inflammatory pacemakers in the very early phase of fracture repair, leading to a delay in skeletogenic mesenchymal stem cell differentiation, with consecutive non- or delayed bone healing (54–56). This finding could serve as one possible explanation why polytraumatised patients suffer from bony non-union more often than patients with isolated injury (9, 30, 57). Against this background, the value of traumatic hemorrhage and its influence on the local immunologic milieu in fracture healing must not be underestimated. Comparable to the benefit of typical shock organs (58), it seems likely, that early resuscitation would also improve the perfusion at the site of the fracture zone supporting recovery to a physiological and immunological state (59, 60). In this context, Augat et al. found that a transient hemorrhagic shock situation followed by isovolumetric blood volume resuscitation resulted in improved fracture healing. The authors concluded that the positive healing response might be associated with improved revascularization of the soft callus adjacent to the fracture site (59, 60). Accordingly, Melnyk et al. described that soft tissue damage without destruction of the bone-soft tissue interface is likely to have only a limited effect on fracture healing (61).

Beside its pro-inflammatory properties, IL-8 is well-known for its angiogenetic characteristics. Accordingly, high local levels were found in fracture haematoma in a previous clinical study, which underlines the importance of IL-8 in the process of bone healing (28). Our data revealed that IL-8 kinetics in muscle tissue showed opposite trends to those seen in fracture haematoma. While concentrations in muscle tissue increased initially and decreased during the clinical course, haematoma concentrations initially decreased and recovered during the later clinical course. Comparable to the observations made in IL-6, the dynamics of IL-8 in fracture haematoma may be explained by reduced blood flow due to haemorrhagic shock in PT (62). Accordingly, Heppenstall et al. report on a rabbit model with an inhibition of fracture healing in hypovolaemia, which was attributed to impaired delivery of oxygen to the fracture site (63). According to our divergent findings in muscle tissue and fracture haematoma, Schmidt-Bleek et al. reported differences in the immunologic milieus of muscle haematoma and fracture haematoma in a sheep model (64). The authors indicate that the inflammatory processes differ due to a unique immune cell composition (64). Although the authors report on a different animal model of isolated trauma, investigating cell migration, our data also reveal differences in the immunologic post-traumatic milieus of muscle tissue and fracture haematoma within the MT, but not the PT group. In regard to the concentrations measured in muscle tissue, our findings are supported by Dragu et al. who proved alterations in the gene expression level in human muscle free flaps after ischaemia and reperfusion (65). The authors report on IL8 as one of four genes that were significantly upregulated after reperfusion of ischemic muscle tissue (65). Accordingly, Huda et al. showed that a significantly elevated concentration was measurable in blood plasma after 3–4 h of reperfusion (66). Furthermore, Kukielka et al. investigated IL-8 expression after ischemia and reperfusion in canine myocardium. The authors found that IL-8 mRNA peaked in the first 3 h of reperfusion and persisted at high levels beyond 24 h (67). Based on these findings, Kukielka et al. speculated that surface-bound chemoattractants may represent an effective mechanism of chemotactic agent presentation and neutrophil activation wherever a reduced blood flow prevents the establishment of a stable soluble chemotactic gradient (67). The observation of increased IL-8 levels are in line with our results from muscle tissue analysis. Both groups showed increased IL-8 concentrations 24 and 48 h after trauma. While values in MT were doubled, concentrations in PT increased even four times compared to initial values. As PT received haemorrhagic shock, this finding might support Kukielka's speculation on the effect of surface-bound chemoattractants in tissue with reduced blood flow. Thus, cell composition as well as interaction of immunologic key players in the early local inflammatory response after multiple trauma must be the focus of further studies.

### The Anti-inflammatory Phase

Interleukin-10 is known as an anti-inflammatory mediator that also plays a central role in the fracture healing process (28). It influences bone resorption and enhanced bone healing (35, 68, 69), and a deficit results in osteopenia, mechanical fragility of bones, and defects in their formation (70). While some authors report increased IL-10 concentrations in fracture haematoma during the early post-traumatic phase (71, 72), we could not prove significant kinetics over time. Baker et al. compared different trauma models and proofed that polytrauma plus hemorrhage did not induce the systemic release of IL-10 (49). The authors showed that an additional hemorrhage component appears to attenuate the systemic release of IL-10 after polytrauma (49). In line with Baker et al. and Wichmann et al. proofed that a bone injury, coupled with haemorrhagic shock, produces a more severe depression of immune functions than a haemorrhagic shock alone (73). The authors concluded that bone injury appears to play a contributory role in further depressing immune functions in trauma patients who experienced major blood loss (73). These observations may further reflect that the combined insult leads to the induction of a state of immune paralysis, which also affects IL-10 concentrations within the fracture haematoma (49, 74). In contrast, Hauser et al. found significantly increased IL-10 levels in fracture haematomas in the early phase after trauma, whereas lower levels were observed in the later period (>24 h) (71). However, the authors reported on isolated injures with very heterogeneous entity and severity, which might not realistically reflect the polytraumatised situation. Additionally, Hoff et al. also reported elevated IL-10 concentrations in fracture haematoma (72). However, these values were compared to IL-10 concentrations gained from non-traumatic osteotomy in hip replacement. Thus, the expressiveness of this early “increase” might be also questioned against the background of traumatic injuries. Delayed migration of IL-10-producing cells into fracture haematoma, proved by Schmidt-Bleek et al. may be another cause for time-dependent kinetics in local IL-10 concentrations (44). This might allow a careful speculation about the observed IL-10 increase in fracture haematoma of the MT group, but not the PT group after 72 h, representing a possible shift from a pro-inflammatory immunologic milieu toward an anti-inflammatory and angiogenic one (28, 44, 75). Yet, literature about local concentrations of IL-10 remains sparse, and further research is warranted. However, the absence of IL-10 in the haematoma of polytraumatised patients might be another explanation for impaired bone regeneration in this patient cohort.

### Limitations

The purpose of our study was to gain knowledge about trauma impact and its effect on local inflammatory response around the fracture zone in a clinically relevant, large animal model of isolated vs. multiple trauma. Unfortunately, molecular mechanisms that regulate local or systemic inflammatory response could not be derived. Also, interaction of local inflammatory response to osteo- and chondrogenesis remain unlighted. Furthermore, testing of a relatively small sample size yielded relatively large standard errors for each parameter. Additionally, it would have been interesting to analyse individual immunologic responses as well as financial restrictions led to measurement of only three mediators which is regrettable in the context of a vast immunologic system whereby dozens of inflammatory mediators dynamically interact resulting in a plethora of possible phenotypes. However, research regarding this field is ongoing, and follow-up studies that concentrate on cell migration, but also on bone healing, are in preparation.

## Conclusions

To the best of our knowledge, this is the first study that characterizes and compares chronologic data of locally active inflammatory mediators in regard to femur fracture and trauma impact. Although inference of systemically circulating mediators cannot be drawn from this study, it might be suggested that concomitant injuries, such as haemorrhagic shock, significantly influence local post-traumatic reactions in fracture/soft-tissue haematomas. Combined trauma (or “severe trauma”) may cause perturbations in local and/or systemic cytokines and chemokine levels intimately involved in the early phases fracture healing, which may influence adverse outcomes such as fracture non-union. Based on the results of this study, further studies of our group will focus on the role of inflammatory mediators in the repairing process of injured tissue and their role in the systemic process of responding to trauma.

## Advances in Knowledge

To our knowledge, this is the first study comparing and discussing local (fracture haematoma and muscle tissue) inflammatory response in a large animal model of isolated (MT) and combined (PT) trauma, giving chronological data of locally active inflammatory mediators in regard to extremity fracture during early post-traumatic stages up to 72 h.

## Ethics Statement

This study was carried out in accordance with the recommendations of the German legislation governing animal studies, following The Principles of Laboratory Animal Care (16). Official permission was granted from the governmental animal care and use office (Landesamt für Natur, Umwelt und Verbraucherschutz Nordrhein-Westfalen, Recklinghausen, Germany, AZ: 84.02.04.2014A265). The protocol was approved by the governmental animal care and use office (Landesamt für Natur, Umwelt und Verbraucherschutz Nordrhein-Westfalen, Recklinghausen, Germany, AZ: 84.02.04.2014A265).

## Author Contributions

KH and FH conceived the study, set up its design and coordinated the experimental and analytic phase. KH, JG, HL, QZ, and TS carried out the experiments and performed the analysis. RP participated in its design and coordination. BR, IM, and H-CP conceived the study, participated in its design and coordination, and helped to draft the manuscript. All authors listed have made a substantial, direct and intellectual contribution to the work, read and approved the final manuscript.

### Conflict of Interest

The authors declare that the research was conducted in the absence of any commercial or financial relationships that could be construed as a potential conflict of interest.
